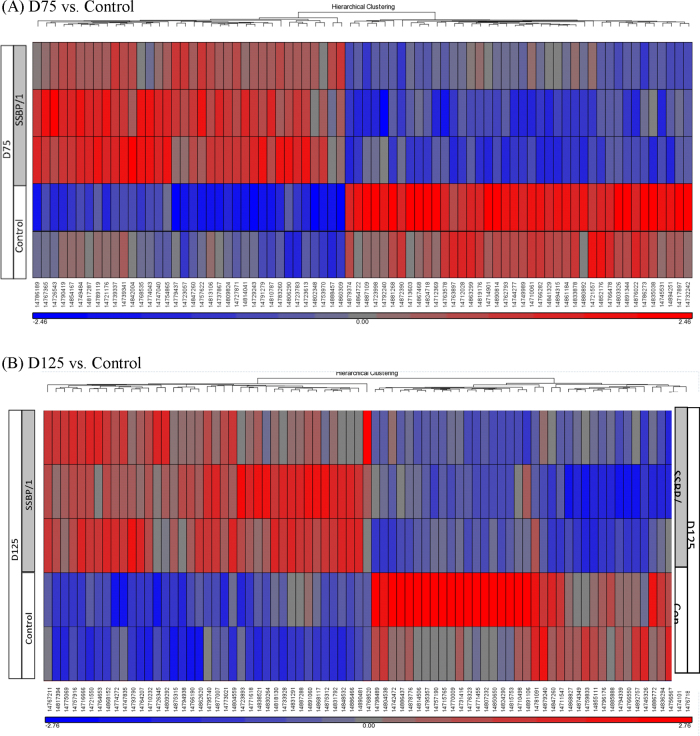# Transcriptome analysis of CNS immediately before and after the detection of PrP^Sc^ in SSBP/1 sheep scrapie

**DOI:** 10.1016/j.vetmic.2014.07.026

**Published:** 2014-10-10

**Authors:** Anton G. Gossner, John Hopkins

**Affiliations:** The Roslin Institute & R(D)SVS, University of Edinburgh, Easter Bush, Midlothian, Edinburgh EH25 9RG, UK

**Keywords:** Scrapie, Prion, Gene expression, Thalamus, Medulla, CNS+

## Abstract

Sheep scrapie is a transmissible spongiform encephalopathy (TSE), progressive and fatal neurodegenerative diseases of the central nervous system (CNS) linked to the accumulation of misfolded prion protein, PrP^Sc^. New Zealand Cheviot sheep, homozygous for the VRQ genotype of the *PRNP* gene are most susceptible with an incubation period of 193 days with SSBP/1 scrapie. However, the earliest time point that PrP^Sc^ can be detected in the CNS is 125 days (D125). The aim of this study was to quantify changes to the transcriptome of the thalamus and obex (medulla) at times immediately before (D75) and after (D125) PrP^Sc^ was detected. Affymetrix gene arrays were used to quantify gene expression in the thalamus and Illumina DGE-tag profiling for obex. Ingenuity Pathway Analysis was used to help describe the biological processes of scrapie pathology.

Neurological disease and Cancer were common Bio Functions in each tissue at D75; inflammation and cell death were major processes at D125. Several neurological receptors were significantly increased at D75 (e.g. *CHRNA6*, *GRM1*, *HCN2*), which might be clues to the molecular basis of psychiatric changes associated with TSEs. No genes were significantly differentially expressed at both D75 and D125 and there was no progression of events from earlier to later time points. This implies that there is no simple linear progression of pathological or molecular events. There seems to be a step-change between D75 and D125, correlating with the detection of PrP^Sc^, resulting in the involvement of different pathological processes in later TSE disease.

## Highlights

• Arrays and DGE-tags quantified gene expression in the CNS during sheep scrapie.
• Neurological receptors were increased with disease progression.
• Clues to basis of psychiatric changes.
• Step changes to gene expression after the detection of PrP^Sc^ in CNS.

## 1 Introduction

Sheep scrapie is a transmissible spongiform encephalopathy (TSEs); fatal diseases of the central nervous system (CNS) that also includes Creutzfeldt Jakob Disease (CJD) in humans, chronic wasting disease in deer and bovine spongiform encephalopathy (BSE). The principal pathological features of these diseases are neural cell degeneration, spongiform change and glial cell hypertrophy (Prusiner et al., 1998) that are linked to the conversion of the normal cellular prion protein (PrP^C^, encoded by the *PRNP* gene) to the disease-associated conformer PrP^Sc^ (Prusiner, 1982). The central role of PrP^C^ in scrapie is illustrated by the fact that *PRNP* knockout mice are resistant to disease (Bueler et al., 1993) and by the reciprocal relationship between *PRNP* copy number and incubation period (Bueler et al., 1993). In addition, susceptibility to scrapie in sheep is controlled by *PRNP* genotype (Goldmann et al., 1994) at codons 136, 154 and 171; in Cheviot sheep ARR/ARR animals are resistant, VRQ homozygotes have the shortest incubation period and heterozygotes are intermediate. The cause of the disease is thought to be linked to replication and accumulation of PrP^Sc^ (Prusiner et al., 1999) and neuronal expression of PrP^C^ is essential for neurodegeneration and the development of disease (Mallucci et al., 2003). However, there are several examples of the lack of correlation of PrP^Sc^ levels in the CNS and disease. In some scrapie strain/host combinations high titres of infectivity are associated with low levels of PrP^Sc^ (Barron et al., 2007); in others the accumulation of PrP^Sc^ in the brain is not associated with transmissible disease (Piccardo et al., 2013). This lack of direct correlation between quantitative levels of PrP^Sc^ aggregation and infectivity raises two major questions. The first is the fundamental question of how scrapie agent/PrP^Sc^ causes cell pathology and disease and secondly, are there surrogate markers of infectivity and disease other than PrP^Sc^.

An understanding of the disease-causing mechanisms of TSE agents is critical for the selection of potential therapeutic targets. In recent years there have been a number of functional genomic studies with the aim of quantifying differential gene expression in the CNS and the consequent identification of molecular pathways associated with the progression of TSE disease. Most of these studies have used mouse models of scrapie (Skinner et al., 2006, Xiang et al., 2004) but more recently BSE and scrapie from orally-infected cattle (Almeida et al., 2011a) and sheep (Filali et al., 2012), and human CJD (Tian et al., 2013) have been examined. Perhaps the most comprehensive analysis was described by Hwang (Hwang et al., 2009) who compared eight combinations of mice and scrapie strains at different time points after infection and identified a panel of differentially-expressed genes central to TSE disease. In addition, similar technologies have been used to try and identify potential alternative markers of TSE infection (Sorensen et al., 2008). The use of a diverse range of species and TSE agents and time points has resulted in a large range of genes and pathways being identified. However, there have been a number of physiological pathways consistently linked to progression of TSE disease in the CNS, including the up-regulation of genes involved in inflammation (Xiang et al., 2004), immune response (Sorensen et al., 2008), autophagy and cell death (Brown et al., 2005) as well as regulation of transcription (Skinner et al., 2006).

Previous studies from this laboratory used the SSBP/1 sheep scrapie model in New Zealand sheep to describe the progression of pathology, as indicated by the detection of PrP^Sc^, in different regions of the brain (Gossner et al., 2011a) and peripheral lymphoid tissues (Gossner et al., 2011b), in sheep with defined *PRNP* genotypes. The earliest time that PrP^Sc^ was detected by immunohistochemistry in the thalamus and obex was 125 days (D125) after subcutaneous inoculation of SSBP/1 in VRQ homozygous animals; terminal disease was 193 ± 12 days. This current study exploits these same sheep to quantify the effects of scrapie infection on the transcriptome of the thalamus and obex immediately before (D75) and at the time point when PrP^Sc^ is consistently detected in the CNS. For these studies we use the new Affymetrix Ovine Gene 1.1 ST whole-genome arrays validated by Illumina digital gene expression-tag profiling (DGS-tag). In this way we examine scrapie infection of the brain and begin to uncouple the effects of infection from the effects of PrP^Sc^ accumulation.

## 2 Materials and methods

### 2.1 Animals and experimental design

Details of sheep, infection regime and tissues have been described previously (Gossner et al., 2011a). Briefly, New Zealand Cheviot sheep homozygous for the *PRNP* genotype VRQ (Houston et al., 2002), were infected subcutaneously in shoulder with SSBP/1 sheep brain homogenate or scrapie-negative brain homogenate. Sheep were in groups of five, 3 scrapie-infected and two uninfected controls and were sacrificed at 75 days (D75) and 125 days (D125) post infection. Animal experiments were approved by BBSRC Institute for Animal Health Ethical Review Committee and conducted under an Animals (Scientific Procedures) Act 1986 Project Licence.

### 2.2 Tissue collection and RNA isolation

Tissues were removed immediately post-mortem, and 5 mm^3^ blocks incubated overnight at 4 °C in RNALater (Ambion, Huntingdon, UK) and stored at −80 °C. Total RNA was isolated using RNeasy Lipid Tissue Mini Kit (Qiagen, Crawley, UK) with DNase I digestion. RNA integrity and quality was assessed by a RNA 6000 Nano LabChip on the Agilent 2100 Bioanalyzer and quantified using the NanoDrop ND-1000 spectrophotometer.

### 2.3 RNA and microarray processing

Sense-strand cDNA from 0.5 μg RNA was amplified using two rounds of the Ambion® WT Expression Kit. Amplified cDNA was biotin labelled and fragmented using the Affymetrix GeneChip® WT Terminal Labelling and Hybridization kit. Labelled cDNA fragments (5.5 μg) were hybridized to Affymetrix Ovine Gene 1.1 ST arrays using the Hyb-Wash-Scan procedure using reagents from the Affymetrix Gene Titan Hyb Wash Stain kit. Plates were then washed, stained and scanned using the Imaging Station of the GeneTitan System. The Affymetrix® GeneChip® Command Console® Software v 3.0.1 was used to generate array images and the Affymetrix CEL files. QC was performed using Affymetrix Expression Console.

### 2.4 Array data analysis

The CEL files were imported into Partek Genomics Suite v 6 (http://www.partek.com/?q=partekgs) and data were analysed at the gene-level, with exons summarized to genes, using the mean expression of all exons of a gene. Background correction used the robust multiarray average (RMA) algorithm, with quantile normalization, median polish probe summarization, and log_2_ prob-e transformation. Differentially expressed genes were identified by analysis of variance and the *q* value, a measure of the proportion of false positives incurred – the false discovery rate, was used as measure of statistical significance (Storey and Tibshirani, 2003). Hierarchical clustering was performed on significant genes, with the data normalized to a mean of zero and scaled to standard deviation of one using Partek Genomics Suite. Significant genes were annotated based on similarity scores in BLASTN comparisons of Affymetrix Transcript cluster sequences against ovine/bovine sequences in GenBank. Detailed protocols and all raw data are available at the ArrayExpress database (www.ebi.ac.uk/arrayexpress) under accession number E-MTAB-2385.

### 2.5 Illumina digital gene expression (DGE) tag-profiling

Sequence tag libraries were constructed using the Illumina DGE Tag Profiling *NlaIII* Sample Prep kit according to the manufacturer's protocol. In brief, mRNA was isolated from 2 μg of total RNA using Oligo dT beads. First- and second-strand cDNA were synthesized while the mRNA was attached to the beads. Subsequently, cDNA samples were digested using *NlaIII*, which recognizes and cuts the most 3′ ‘CATG’ motif. GEX *NlaIII* Adapter 1 was ligated at the site of *NlaIII* cleavage. The GEX Adapter 2 was ligated after *MmeI* digestion. The adapter-ligated cDNA tags were enriched using PCR, and purified on PAGE gel. The purified cDNA tags were used to create clusters on a flow cell using an Illumina Cluster Station and were sequenced on an Illumina Genome Analyser II instrument. Image recognition and base calling were performed using the Illumina Pipeline.

### 2.6 Mapping and analysis of genes identified by DGE-tag profiling

Processed sequence files from the Illumina pipeline output were aligned against version 3.1 assembly of the *Ovis aries* genome (http://www.ncbi.nlm.nih.gov/genome?term=ovis%20aries) with the short read aligner Bowtie (Langmead and Salzberg, 2012). The mapped reads were assembled and annotated into transcript level expression summaries of count data from each sample using the *intersectBed* utility from the BEDTools suite (Quinlan and Hall, 2010) and sheep transcript sequences (http://www.livestockgenomics.csiro.au/sheep/oarv3/Oarv3.1.protein.gene.gff3). The summarized data was filtered and scale normalized before converting the read counts to log_2_-cpm, with associated weights, using the Voom (http://www.statsci.org/smyth/pubs/VoomPreprint.pdf) routine in Limma part of Bioconductor R (http://bioconductor.org). Statistical analysis was performed using the eBayes function after the log-normalized counts were used to construct a linear model and find differentially expressed (DE) genes between SSBP/1 infected and control samples.

### 2.7 Functional enrichment and network analysis

Network analysis was performed with Ingenuity Pathway Analysis software (http://www.ingenuity.com/) to increase confidence in the observations of differentially expressed genes by correlation with biological pathways. This process also allowed the identification of putative key functional elements within the networks of differentially expressed genes. The network interaction of the focused genes in the network is based on their connectivity in Ingenuity Knowledge Base.

## 3 Results

### 3.1 Differential gene expression at D75 and D125

Affymetrix arrays identified 337 significantly differentially-expressed genes with fold change ≥1.5 and adjusted *p* value (*q* value) ≤ 0.05 at D75 after SSBP/1 infection, prior to the immunohistochemical detection of PrP^Sc^ (Gossner et al., 2011a) in the thalamus (Table S1), in comparison to uninfected controls (C); 163 genes were significantly increased and 174 were repressed. At D125, the first time point when PrP^Sc^ was detected in the thalamus, 243 genes were significantly differentially-expressed, with 162 genes increased and 81 repressed (Table S2). No genes were common in the two comparisons D75 vs. C and D125 vs. C; neither were there any significantly expressed genes in the direct comparison of the two infected groups, D75 vs. D125. Presentation of these data by heat map (Fig. S1) illustrates the consistency of the data from the individual sheep within the infected and control groups.

The most up-regulated genes in the infected thalamus (Table 1) included *CHRNA3* (+5.81 fold), *MET* (+3.81 fold) and *MEIS2* (+2.99 fold) at D75; and *PARP3* (+3.17 fold) and *CAMP* (+2.50 fold) at D125. The most repressed included *HP* (−5.22 fold) and *AVP* (−6.59 fold) both *q* ≤ 0.0497; and *CD3E* (−4.62 fold), *CCR7* (−5.65 fold) and *IGHV4-61* (−9.82 fold) at D125. The array data were also analysed in relation to fold change but without regard to p value (Table S3) helping to confirm the data in Table 1 and Tables S1 and S2. The top-ranked gene at D75 was also a neuronal cholinergic receptor, (*CHRNA2*, +7.43 fold, *p* = 0.1689); *CHRNA5* was also increased 3.09 fold (*p* = 0.0755) at this D75 time point. Many of the most repressed genes at D125 (but *q* > 0.05) are associated with lymphocyte function. The B cell receptor gene *CD72A* was decreased (+3.06 fold, *p* = 0.0917) as were the immunoglobulin heavy chain genes, *IGHE* (−7.93 fold, *p* = 0.1142), *IGHA2* (−6.14 fold, *p* = 0.0922), IGM (−5.97, *p* = 0.0744) and both light chain genes, IGK (−4.24, *p* = 0.1346) and IGL (−3.85, *p* = 0.0576). Genes associated with the T cell receptor/CD3 complex were also repressed, including *TRB* −2.43 fold, *p* = 0.2296), *CD3D* (−4.39 fold, *p* = 0.0869) and *CD3G* (−2.97 fold, *p* = 0.1245). Raw data as well as detailed protocols of the experimental procedures, methods of analysis and array data are available as supplementary information in the ArrayExpress database (www.ebi.ac.uk/arrayexpress) under accession numbers E-MTAB-2369 (array) and E-MTAB-2385 (DEG-tag).

**Table 1:** Top five up- and down-regulated genes at D75 and at D125.

Transcript	Gene symbol	Accession no	Gene name	*q* Value	FC
Genes significant at D75					
14817287	CHRNA3	XM_004017821.1	Cholinergic receptor, nicotinic, alpha 3	0.0497	5.81
14727871	HAPLN4	XM_004009142.1	Hyaluronan and proteoglycan link protein 4	0.0497	4.28
14721176	MET	NM_001111071.1	*met* Proto-oncogene	0.0497	3.81
14739337	MALRD1	XM_005890186.1	MAM and LDL rec class A domain containing 1	0.0497	3.38
14723057	MEIS2	XM_004023192.1	Meis homeobox 2	0.0497	2.99
14714901	HP	XM_004015111.1	Haptoglobin	0.0497	−5.22
14841329	SIM1	XM_004011247.1	Single-minded homolog 1	0.0497	−5.23
14819176	GRP	NM_001009321.1	Gastrin-releasing peptide	0.0497	−5.68
14861184	FOXB2	XM_002689669.2	Forkhead box B2	0.0497	−6.00
14894315	AVP	NM_001126341.1	Arginine vasopressin	0.0497	−6.59
Genes significant at D125					
14795740	PARP3	XM_004018417.1	Poly (ADP-ribose) polymerase family, member 3	0.0498	3.17
14716666	CLHC1	XM_004005946.1	Clathrin heavy chain linker domain containing	0.0429	2.98
14766190	CAMP	U60598.1	Cathelicidin antimicrobial peptide	0.0459	2.50
14848532	FZD7	XM_004005406.1	Frizzled family receptor 7	0.0474	2.48
14817394	PPOX	XR_173400.1	Protoporphyrinogen oxidase	0.0459	2.44
14781091	HLA-A	XR_173400.1	Major histocompatibility complex, class I, A	0.0474	−4.44
14715765	CD3E	NM_001009418.2	CD3e molecule, epsilon (CD3-TCR complex)	0.0498	−4.62
14795357	HIST1H1B	XM_605694.3	Histone cluster 1, H1b	0.0498	−5.37
14757190	CCR7	XM_004012868.1	Chemokine (C–C motif) receptor 7	0.0498	−5.65
14814506	IGHV4-61	AJ505116.1	Immunoglobulin heavy variable 4–61	0.0498	−9.82

The five most up- and down-regulated genes based on fold change (*q* ≤ 0.05) in SSBP/1-infected thalamus at D75 and at D125 as assessed by Affymetrix whole genome array.

### 3.2 Ingenuity pathway analysis

Ingenuity pathway analysis (IPA) was used to help describe how differentially-expressed genes influence the biological processes leading to the development of CNS pathology in the SSBP/1 model of sheep scrapie. The Affymetrix ovine gene arrays identified eight networks (*P*-scores < 26) in the D75 and D125 vs. C comparisons (Table 2). The two top-ranked networks were ‘Hereditary disorder, metabolic disease, cancer’ at D75 and ‘Dermatological diseases and conditions, developmental disorder, organismal injury and abnormalities’ at D125, both with *P*-scores of 37. The top Bio Functions within these networks at D75 (Table 3) were ‘Neurological disease’ with 47 genes with the highest *p* value of 1.37 × 10^−6^ and ‘Cancer’ with 72 genes 5.06 × 10^−6^ associated with Diseases and Disorders, and ‘Cell morphology’ with 25 genes *p* = 4.52 × 10^−7^ (Molecular and cellular functions). Also notable were ‘Psychological disorders’ with 28 genes *p* = 3.41 × 10^−5^ (Diseases and disorders) and ‘Cell death and survival’ with 42 genes *p* = 2.58 × 10^−6^ (Molecular and cellular functions).

**Table 2:** Top networks identified by Ingenuity Pathway Analysis from Affymetrix whole genome array.

Thalamus D75 vs. C	*P*-score
Hereditary disorder, metabolic disease, cancer	37
Cellular movement, molecular transport, cancer	32
Cell cycle, auditory disease, auditory and vestibular system development and function	31
Drug metabolism, small molecule biochemistry, organismal injury and abnormalities	27
Cell-to-cell signaling and interaction, hematological system development and function, humoral immune response	26

Thalamus D125 vs. C	*P*-score
Dermatological diseases and conditions, developmental disorder, organismal injury and abnormalities	37
Cell morphology, hematological system development and function, cell-mediated immune response	35
Cell-to-cell signaling and interaction, cellular growth and proliferation, cell morphology	26

**Table 3:** Top Bio Functions identified by IPA from Affymetrix whole genome array.

	*P*-value	No of molecules
D75 vs. C		
Diseases and disorders		
Neurological disease	1.37E-06–1.19-02	47
Cancer	5.06E-06–1.19-02	72
Psychological disorders	3.41E-05–1.13-02	28
Molecular and cellular functions		
Cell morphology	4.52E-07–1.19-02	25
Cellular function and maintenance	4.52E-07–1.19-02	38
Cell death and survival	2.58E-06–1.19-02	42
D125 vs. C		
Diseases and disorders		
Immunological disease	3.10E-04–3.53E-02	17
Inflammatory response	2.36E-04–4.29E-02	16
Cancer	4.02E-04–4.33E-02	20
Molecular and cellular functions		
Cell death and survival	3.53E-06–3.02E-02	22
Cell growth and proliferation	3.85E-06–4.00E-02	22
Cell morphology	5.16E-06–4.00E-02	14

At D125 top Bio Functions include (Table 3) ‘Immunological disease’ with 17 genes *p* = 3.10 × 10^−4^ (Diseases and Disorders) and ‘Cell death and survival’ with 22 genes *p* = 3.52 × 10^−6^ (Molecular and cellular functions). Also notable were ‘Inflammatory response’ with 16 genes *p* = 2.36 × 10^−4^, and ‘Cancer’ with 20 genes *p* = 4.02 × 10^−4^ (Diseases and disorders) and ‘Cell growth and proliferation’ with 22 genes *p* = 3.85 × 10^−6^ (Molecular and cellular functions). The identity of the genes associated with the Bio Functions is shown in Table S4A).

### 3.3 DGE-tag profiling

Illumina Solexa DGE-tag profiling was performed on the obex (medulla) of the same animals as used for the Affymetrix arrays and delivered a mean of 7,157,973 mer tags per sample (min 3,795,081; max 14,460,672); of which a mean of 532,627 mapped to Oar v3.1. The genes were selected on the basis of log-odds that the gene is differentially expressed, or B-statistic (Smyth, 2004). The top 500 genes for each tissue in the obex are shown in Table S5. At D75, before PrP^Sc^ could be detected in the obex (Gossner et al., 2011a), 278 genes were increased and 223 repressed. D125 was earliest time point that PrP^Sc^ was detected, and 222 were increased and 278 repressed. Of the 500 genes in each comparison, 360 genes (203 increased and 157 repressed) with *p* ≤ 0.05 and 206 with *q* ≤ 0.05 at D75; at D125 there were 311 (138 increased and 173 repressed) with *p* ≤ 0.05 but none with *q* ≤ 0.05, the lowest *q* value was 0.0786.

IPA analysis of the DGE-tag dataset for the two time points for the obex is shown in Table 4. Comparison of this analysis with the IPA from the Affymetrix array data for the thalamus at D75 (Table 3) identified the ‘Cancer’ Bio-Function in common in Diseases and Disorders, and ‘Cell movement’ and ‘Cellular function and maintenance’ in Molecular and Cellular Functions. At D125 ‘Cancer’ is also a common Bio-Function in Diseases and Disorders; and in Molecular and Cellular Functions all three Bio-Functions are common to both analyses, ‘Cell Death and Survival’, ‘Cell Morphology’ and ‘Cell Growth and Proliferation’. The identity of the genes associated with the Bio Functions is shown in Table S4B.

**Table 4:** Top Bio Functions at D75 and D125 identified by IPA from DGE-tag analysis.

	*P*-value	No of molecules
D75 vs. C		
Diseases and disorders		
Cancer	6.83E-06–2.23E-02	110
Organismal injury and abnormalities	7.89E-05–2.23E-02	29
Neurological disease	4.06E-04–2.03E-02	69
Molecular and cellular functions		
Cellular movement	1.58E-04–1.90E-02	22
Cell assembly and organization	2.56E-04–2.07E-02	38
Cellular function and maintenance	6.25E-04–1.99E-02	36
D125 vs. C		
Diseases and disorders		
Cancer	7.35E-06–1.43E-02	193
Neurological disease	4.04E-04–1.43E-02	42
Organismal injury and abnormalities	5.83E-04–1.43E-02	54
Molecular and cellular functions		
Cell death and survival	9.70E-05–1.43E-02	88
Cell morphology	1.72E-04–1.43E-02	61
Cell growth and proliferation	2.04E-04–1.43E-02	39

## 4 Discussion

This project is a development of a previous RT-qPCR study on the expression of selected genes in different regions of the CNS during the time course of SSBP/1 scrapie infection (Gossner et al., 2011a), and aims to detail changes in the transcriptome before, and immediately after the detection of PrP^Sc^ in the thalamus and obex (medulla) regions of the CNS. The thalamus and obex are both sites of scrapie pathology (González et al., 2003) and PrP^Sc^ aggregation can be detected for the first time in both tissues at D125 (Gossner et al., 2011a). Transcriptome analysis of the thalamus was by the Affymetrix Ovine gene 1.1 ST Array, which consists of probes for all exons in each annotated transcript of the OARv2 sheep genome assembly. Analysis of the obex was by DGE-tag profiling, which provides a more complete coverage of the transcriptome than selected RT-qPCR assays. DGE-tag profiling of obex identified similar numbers of differentially-expressed genes (*p* ≤ 0.05) as the array, with approximately equivalent proportions increased and repressed. However, the array data permitted more accurate annotation than DGE-tag profiling because the tags were only 21 base sequences. The initial RT-qPCR and DGE-profiling exhausted the supply of obex and consequently the thalamus was used for the arrays. Nevertheless, comparable and/or identical IPA bio-functions were identified by both technologies in both tissues.

In view of the fact that the major target organ for TSEs is the brain the majority of gene expression studies on the progression of disease have focussed on the CNS; most of these have used time course analyses in mouse models of scrapie (Skinner et al., 2006, Xiang et al., 2004). A difference between these time course studies in mice and this study is that SSBP/1 sheep scrapie does not show a simple linear progression, but rather a step-wise change from D75 to D125, relating to the times when PrP^Sc^ can be detected by immunohistochemistry.

All the mouse studies identified a large range of differentially-expressed genes, but most showed consistent reduction in the genes associated with cholesterol metabolism and promoting complement fixation, inflammation and cell death/apoptosis. A large, multi-component study using two mouse-adapted scrapie strains infecting five mouse strains over several time points, identified a core set of 333 genes that appear central to prion disease (Hwang et al., 2009). It also identified similar physiological networks to the previous studies. Our study identified 33 of this core set that were differentially-expressed, including *CXCR4*, *S100A11* and *NCF3* at D75 and *VCAM1*, *XCL1* and *ADAM4* at D125. The current study also highlights inflammation and apoptosis/cell death as significant components of TSE pathogenesis, although this is particularly true at the D125 time point (e.g. *CAMP* +2.5 fold and *TRADD* +1.77 fold). However, a major difference is that there seems to be no reduction in cholesterol metabolism genes in the sheep study. A possible reason for this could be one of species difference as studies of bovine BSE (Almeida et al., 2011b) also fail to identify a reduction in cholesterol associated genes.

Two of the major pathological features of TSEs are microglial activation and astrocyte proliferation (Rezaie and Lantos, 2001), and this may explain that Ingenuity Pathway Analysis for both the array and DGE-tag datasets highlights Bio Functions associated with inflammation, immunity and cancer. Inflammatory infiltration and polymorphonuclear cell cuffing in the blood vessels of the brain are not features of TSE disease (Porter et al., 1973). Indeed, the data reported here indicate a marked reduction in lymphocyte infiltration into the diseased brain, especially at D125, illustrated by repression of many genes associated with both lymphocyte populations, including B cell (e.g. *IGHV*, *CD79A*) and T cell receptor complexes (e.g. *TRA*, *CD3E*). There is also significant repression of *XCL1* and *CCR7* (and *CCL21*, *p* > 0.05), chemokine/receptors that control lymphocyte but not myeloid cell migration (Rot and von Andrian, 2004). The increase in inflammatory gene expression was seen largely at D125 after the detection of PrP^Sc^; this is consistent with other studies that describe the production of inflammatory mediators by glia (Peyrin et al., 1999), possibly activated by fragments of PrP^Sc^ (Rezaie and Lantos, 2001).

The other major pathological feature of all TSEs is neurodegeneration, and the ‘Neurological disease’ Bio Function is identified in the thalamus at D75 and in the obex at both D75 and D125. Two highly repressed genes within this Bio Function play major roles in the regulation of blood pressure; *AGTR1* (−4.57 fold) and *AVP* (−6.59 fold) are both vasoconstrictors and low expression levels in the brain reduces blood supply (Matsusaka and Ichikawa, 1997) leading to impaired memory and cognition as well as social dysfunction (Bielsky et al., 2004). Furthermore, modifications to the interactions between arginine vasopressin (*AVP*) and its receptor results in subtle olfactory deficits, evidenced by 6 olfactory genes significantly increased (e.g. *OR6C4* +2.03 fold) and 8 repressed (e.g. *OR8B3* −1.80 fold) at D75, and 18 genes significantly increased and 2 repressed at D125.

The nicotinic acetylcholine receptor family proteins are ion-channels that play a role in neurotransmission and are linked to nicotine and alcohol dependence (Exley et al., 2011). In the CNS they are expressed by the dopaminergic neurons and differential expression can lead to anxiety and neuroses followed by depression (Hogg et al., 2003). In this study *CHRNA3* and *CHRNA6* are significantly raised at D75 (+5.81 and +2.47 fold); and three other members of the family are non-significantly increased, *CHRNA2* (+7.47 fold), *CHRNA4* (+2.04 fold) and *CHRNA5* (+3.09 fold). No acetylcholine receptors are significantly differentially-expressed at D125 but *CHRNA2* (−2.99 fold) and *CHRNA3* (−2.40 fold) are repressed but *p* > 0.05. Two other neuronal receptors are also differentially expressed at D75. *GRM1* is associated with a number of neurological diseases including schizophrenia and depression (Kohara et al., 2008) and is significantly increased +1.83 fold, and *HCN2*, which contributes to spontaneous brain rhythmic activity, and dysfunction is associated with epilepsy (Bender et al., 2003), is increased +2.7 fold. These data imply that alterations in expression of these genes may play a role in the psychiatric changes associated with TSEs.

In conclusion, SSBP/1 sheep scrapie infection in its natural host identifies changes to the transcriptome of the thalamus and obex at times immediately before and after the detection of the disease-associated form of the prion protein, PrP^Sc^. The analysis identifies some of the molecular mechanisms associated with TSE disease in sheep and highlights: inflammation, possibly linked to astrocyte activation by PrP^Sc^ fragments; cell death and survival possibly associated with neurodegeneration; and cancer, possible linked to gliosis. In addition, the identity of some of the genes within the ‘Neurological disease’ Bio Function may give clues to the basis of TSE-associated psychiatric problems. However, there seems to be no relationship and little progression of quantitative gene changes, from before (D75) to after the detection of PrP^Sc^ (D125). These data imply that SSBP/1 sheep scrapie is not a simple linear progression from infection to death, but that the eventual accumulation of disease-associated PrP^Sc^ triggers a step change in CNS and consequently a change in the pathological process.

## Competing interests

The authors declare that they have no competing interests.

## Figures and Tables

**Figure:**