# Supplementary material for: Transcriptome analysis of CNS immediately before and after the detection of PrPSc in SSBP/1 sheep scrapie
Source: Vet Microbiol. 2014 Oct 10;173(3-4):201–7. doi: 10.1016/j.vetmic.2014.07.026 (PMC4206282; doi:10.1016/j.vetmic.2014.07.026)
Supplement: Supplementary file 4 [file mmc4.docx]

**Table S4**

Top Bio Functions at (A) D75 and (B) D125 identified by IPA from Affymetrix whole genome array

**A D75 vs. Control**

| **Diseases and disorders** | **P-value** |
| --- | --- |
| **Neurological disease** | 1.37E-06 - 1.19-02 |
| ACTR3B,ADIPOQ,AGTR1,ANXA1,AVP,BMP6,C4A/C4B,CD99,CHEK2,CHRNA6, CLRN1,COX7A2,CRB1,CRYM,CSRP2,CXCR4,FRZB,GCLC,GRM1,HCN2,HNRNPA1,HP,HSD17B6,ID3,IGFBP2,ITGB4,LC16A9,MEIS2,MET,NCS1,NDUFS6,NMB,NR4A1,PDE11A,PIK3R4,PPP1R1B,RDH12,RFX4,S1PR3,SCN1A,SEMA3F,SFRP2,SLC16A2, SLC16A9,SLC2A3,TGIF1,TINAGL1 |  |
| **Cancer** | 5.06E-06 - 1.19-02 |
| A2ML1,ABI3BP,ACTR3B,ADIPOQ,AGTR1,ALDH1A3,ANXA1,ASAH2,ATP2B4,AVP, BCAT1,BLNK,BMP6,C4A/C4B,CD99,CHEK2,CHRNA3,CHRNA6,CNTN6,CPNE2, CRB1,CSRP2,CXCR4,DUSP9,FAM19A2,FHAD1,FRZB,GALNTL6,GCNT4,GEM, GRM1,GRP,GULP1,HAPLN4,HEPACAM2,HNRNPA1,HP,HS3ST4,HSD17B6,ID3, IGFBP2,ITGB4,KCTD8,KLHDC8A,LRIG3,LRTM2,MET,NMB,NR4A1,NTS,PDE11A,PDLIM4,PIK3R4,PLD2,PPP1R1B,PXDN,RDH12,S100A11,SCN1A,SEMA3F,SFRP2,SIM1,SLC16A2,SLC16A9,SLC2A3,SNX7,TGIF1,TMC5,TMEM229B,TTK,WDR49,ZC2HC1C |  |
| **Psychological disorders** | 3.41E-05 - 1.13-02 |
| AGTR1,AVP,C4A/C4B,CHRNA3,CHRNA6,COX7A2,CRB1,CRYM,CSRP2,GCLC, GRM1,HP,HSD17B6,ID3,IGFBP2,ITGB4,MEIS2,MET,NCS1,NR4A1,PDE11A, PPP1R1B,RDH12,RFX4,SCN1A,SCN1A,SEMA3F,SLC16A9 |  |
| **Molecular and cellular functions** | **P-value** |
| **Cell morphology** | 4.52E-07 - 1.19-02 |
| ADIPOQ,AGTR1,ANXA1,AVP,BMP6,CHRNA3,CLRN1,CRB1,CXCR4,GEM,GRM1, GRP,ID3,IGFBP2,ISL1,ITGB4,MET,NMB,NR4A1,NTS,OTOS,PLD2,REM2,S1PR3, TGIF1 |  |
| **Cellular function and maintenance** | 4.52E-07 - 1.19-02 |
| ADIPOQ,AGTR1,ANXA1,ATP2B4,AVP,BMP6,C4A/C4B,CD99,CHEK2,CHRNA3, CHRNA6,CLRN1,CXCR4,DUSP9,GEM,GRM1,GRP,HCN2,ID3,ITGB4,MET,NCS1, NMB,NR4A1,NTS,PLD2,PPP1R1B,RAB27A,RDH12,REM2,S1PR3,SCN1A,SIM1, SLC2A3,TGIF1,TRHR,TTK,UMOD |  |
| **Cell death and survival** | 2.58E-06 - 1.19-02 |
| ADIPOQ,AGTR1,ALDH1A3,ANXA1,ASAH2,ATP2B4,AVP,BLNK,BMP6,CD99,CHEK2,CXCR4,DUSP9,FRZB,GCLC,GEM,GRM1,GULP1,HNRNPA1,HP,ID3,IGFBP2,ISL1, ITGB4,MET,NCS1,NR4A1,NTS,PDLIM4,PLCD3,PLD2,PPP1R1B,RAB27A,RDH12, S100A11,S1PR3,SEMA3F,SFRP2,SLC2A3,SNX7,TTK,UMOD |  |

**B D125 vs. Control**

| **Diseases and disorders** | **P-value** |
| --- | --- |
| **Immunological disease** | 3.10E-04 - 3.53E-02 |
| CD3E,KIAA0101,LCK,TRA,TRB,ADAMTS4,CAMP,CCR7,CD6,CYP2B6,HNRNPA1,PDYN,POU5F1,TNFAIP3,XCL1,DUSP10,IFI30,MLP |  |
| **Inflammatory response** | 2.36E-04 - 4.29E-02 |
| ANGPTL2,CA14,CAMP,CCR7,CD3E,CD6,DUSP10,IFI30,LCK,PDYN,SPIC,TNFAIP3, TRA,TRADD,TRB,XCL1, |  |
| **Cancer** | 4.02E-04 - 4.33E-02 |
| LCK,TRA,CCR7,TNFAIP3,TRB,STK31,CD3E,ANGPTL2,FZD7,MLPH,CDCP1,ADAMTS4,KIAA0101,POU5F1,CAMP,CYP2B6,HNRNPA1,MEOX2,NEK2,PRR15L |  |
| **Molecular and cellular functions** | **P-value** |
| **Cell death and survival** | 3.53E-06 - 3.02E-02 |
| CAMP,CCR7,CD3E,CD6,CDCP1,CYP2B6,DKK3,DUSP10,HNRNPA1,KIAA0101,LCK,MEOX2,NCS1,NEK2,PDYN,POU5F1,STK31,TNFAIP3,TRA,TRADD,TRB,XCL1 |  |
| **Cell growth and proliferation** | 3.85E-06 - 4.00E-02 |
| ADAMTS4,CAMP,CCR7,CD3E,CD6,CDCP1,CYP2B6,DKK3,DUSP10,FZD7,HNRNPA1,IFI30,KIAA0101,LCK,NEK2,POU5F1, SKAP1,TNFAIP3,TRA,TRADD,TRB,XCL1 |  |
| **Cell morphology** | 5.16E-06 - 4.00E-02 |
| CD3E,LCK,TRA,TRB,KIAA0101,CCR7,SPIC,NEK2,CAMP,TNFAIP3,ARRDC2,INF2,PTPN20A/PTPN20B,DKK3 |  |
